# Frequency of Human Papillomavirus infection in squamous cell carcinoma of the oral cavity and oropharynx

**DOI:** 10.1186/1753-6561-8-S4-P67

**Published:** 2014-10-01

**Authors:** Priscila Marinho de Abreu, Pedro Leite Azevedo, Anna Clara Gregório Có, Isabella Bittencourt do Valle, José Roberto Vasconcelos de Podestá, Melissa de Freitas Cordeiro-Silva, Sônia Alves Gouvea, Sandra Lucia Ventorin von Zeidler

**Affiliations:** 1 Federal University of Espírito Santo, Post Graduate Program in Biotechnology, Health Sciences Center - Maruípe, 29040-090, Vitória, Espirito Santo, Brazil; 2 Federal University of Espírito Santo, Department of Pathology, Health Sciences Center - Maruípe, 29040-090, Vitória, Espirito Santo, Brazil; 3 Federal University of Espírito Santo, Department of Physiological Sciences, Health Sciences Center - Maruípe, 29040-090, Vitoria, ES, Brazil

## Background

Squamous cell carcinoma of the oral cavity and oropharynx is a public health issue, with over 200,000 new cases worldwide each year [1]. Studies show that the incidence is higher in male individuals, aged between 40 and 55 years. The consumption of tobacco and alcohol are well-established risk factors for developing head and neck cancer, and studies have been shown synergism between these substances, which contributes to the increased risk [4]. Recently, persistent infection with high risk Human Papillomavirus (HPV) has been identified as a potential risk factor for the development of these tumors [2]. HPV is a DNA virus that features tropism to epithelial cells. The high risk HPV types, such as types 16 and 18 have the ability to integrate their DNA into the host cell DNA, immortalizing keratinocytes, with great possibilities of causing a cancer by infecting cells [3]. This study aims to evaluate the frequency of HPV infection, smoking and alcohol consumption related to the development of squamous cell carcinomas of the oral cavity and oropharynx.

## Methods

99 frozen tumors samples, classified as squamous cell carcinoma of the oral cavity (n = 83) and the oropharynx (n = 16) were analysed. The polymerase chain reaction was performed from the DNA of the samples, using the Human β-globin genes. After, electrophoresis was performed in 1% agarose gel containing ethidium bromide, to confirm the amplification and quality of extracted DNA, then the DNA samples were subjected to nested PCR with primers MY09/11 and GP5 + / GP6 + for viral DNA detection of HPV subtypes 16 and 18. Samples known to be positive for both subtypes of HPV 16 and 18 were used positive controls. Subsequently, the amplified samples were submitted to electrophoresis on a polyacrylamide gel. History of exposure to tobacco and alcohol was investigated through interviews.

## Results and conclusions

Patients with tumors were mostly male (80.81%) aged 30-93 years (average 57.6 years). The use of tobacco was observed in 84.8% of cases and the alcohol abuse of 77.7%. HPV infection was detected in 4 of 99 samples, which corresponds to a low prevalence (4.04%). All of the HPV positive cases also presented the historical of tobacco and alcohol use, that represented about 74.74% of the patients. Studies on the frequency of HPV in Brazilian subjects with head and neck tumors has shown variation 0-33% positivity. [5] Therefore, it is not possible affirm that HPV infection is the main cause of squamous cell carcinoma in these patients, since there is patients that consume alcohol and tobacco concomitantly, which are also risk factors for developing the disease.

## References

[B1] Pannone G Rodolico V Santoro A Lo Muzio L Franco R Botti G Aquino G Pedicillo MC Cagiano S Campisi G Rubini C Papagerakis S De Rosa G Tornesello ML Buonaguro FM Staibano S Bufo P Evaluation of a combined triple method to detect causative HPV in oral and oropharyngeal squamous cell carcinomas: p16 Immunohistochemistry, Consensus PCR HPV-DNA, and In Situ Hybridization Infect Agents and Cancer 2012 7 4 22376902 10.1186/1750-9378-7-4 PMC3313884

[B2] Boscolo-Rizzo P Del Mistro A Bussu F Lupato V Baboci L Almadori G Da Mosto MC Paludetti G New insights into human papillomavirus-associated head and neck squamous cell carcinoma Acta Otorhinolaryngol Ital 2013 33 2 77 87 23853396 PMC3665382

[B3] Fraga CAC Oliveira MVM Silveira ACO Souza LR Viana AG De-Paula AMB Guimarães ALS Papilomavírus humano e carcinogênese: uma abordagem molecular da oncogênese viral Unimontes cientifica 2010 12 70 78

[B4] Cognetti DM Weber RS Lai SY Head and Neck Cancer: An Evolving Treatment Paradigm Cancer 2008 113 Suppl 7 1911 1932 18798532 10.1002/cncr.23654 PMC2751600

[B5] Smith EM Rubenstein LM Haugen TH Pawlita M Turek LP Complex etiology underlies risk and survival in head and neck cancer human papillomavirus, tobacco, and alcohol: a case for multifactor disease J Oncol 2012 2012 571862 22315596 10.1155/2012/571862 PMC3270416

